# Engaging grade school learners with an interactive medical imaging activity

**DOI:** 10.1002/acm2.14606

**Published:** 2024-12-16

**Authors:** Jessica M. Fagerstrom, Alyssa C. Alvarez, Ethan O. Cohen, Afua A. Yorke

**Affiliations:** ^1^ Department of Radiation Oncology University of Washington Seattle Washington USA; ^2^ Northwest Medical Physics Center Lynnwood Washington USA

**Keywords:** active learning, community, CT, education, imaging, outreach, teaching

## Abstract

This case report describes a 45‐min active learning lesson plan that engages 4th–5th and 6th–8th grade school students in spatial reasoning through a review of medical imaging. The lesson plan reviews different planar orientations and cross‐sections of computed tomography (CT) images of familiar objects. The lesson is designed to introduce students to the idea that scientists are key contributors to healthcare, including in medical imaging technologies that facilitate the visualization of internal structures of patients without invasive procedures. The lesson demonstrates the three standard anatomical planes, axial, sagittal and coronal, by guiding students through CT image datasets of various objects. Students then are led in an interactive “dissection” of fruit to compare internal structures with medical images. The lesson plan aligns with key aspects of Next Generation Science Standards and aims to spark interest in the field of medical physics among a young student population through an introduction to imaging technologies. Worksheets and imaging datasets are included as supplementary materials to facilitate interested physicists adapting this work for educational purposes in their own communities, with minimal repeated effort.

## 1 INTRODUCTION

Medical physics outreach programs, particularly those targeting grade school students, have the opportunity to spark interest during a critical period in a student's education.^1^ Effective outreach programs utilize active‐learning environments and hands‐on participation to create engaging experiences for students.^2^ These approaches, which incorporate high levels of Bloom's taxonomy of learning (moving beyond recalling of information and basic comprehension into more sophisticated categories of educational goals including applying, analyzing, and evaluating), help students develop a genuine interest in physics.^3^ By showcasing applications of physics in healthcare, such as radiation oncology, diagnostic imaging, nuclear medicine, and radiation safety, medical physics outreach programs have the opportunity to introduce students to a potentially rewarding profession. Investing in community‐based medical physics outreach can promote a bright and inclusive future for the discipline.

This education case report describes a simple standalone educational outreach activity designed to engage 4th–5th grade and 6th–8th grade students in a brief, interactive, and dynamic lesson plan that introduces them to concepts in medical imaging and career opportunities in medical physics. The content also encourages students to develop spatial reasoning and spatial thinking skills, or skills required for visualizing and understanding relationships between objects in space. The lesson uses hands‐on learning and visual aids to make abstract ideas more accessible and to encourage active participation in scientific and technological aspects involved in healthcare. It incorporates low‐cost, widely available materials to allow for implementation in a variety of formal and informal educational settings.

This lesson is aligned with the Next Generation Science Standards (NGSS). These standards are based on work by the National Research Council, the National Science Teachers Association, and the American Association for the Advancement of Science, detail standards in K–12 science education that integrate disciplinary core ideas, science and engineering practices, and crosscutting concepts that bridge scientific and engineering disciplines.^4^ Grade‐level appropriate educational experiences with medical imaging can encourage students to consider structure and function within life sciences or the ideas that “the way in which an object or living thing is shaped and its substructure determine many of its properties and functions.” ^5^ These experiences can also encourage students to engage with ideas of the influence of science, engineering, and technology on patients and within healthcare; understanding that models can be used to simulate or visualize different systems; and identifying and modeling geometric planes by developing possible solutions.

This case report and associated supplementary materials are provided as a resource to medical physics educators who are interested in engaging with community educational outreach and introducing a young audience to foundational concepts in medical imaging and to potential careers in healthcare science.

### 1.1 Learning objectives

General learning objectives were defined for the lesson plan for all students, noting that 6th–8th grade learners will reach more sophisticated levels of sense‐making^6^ for each of these objectives compared to 4th–5th grade learners.

1. Students will use spatial reasoning to consider how medical imaging of different planes and cross‐sections provides varied perspectives on the internal structure of objects. Students will identify the three main anatomical planes (sagittal, coronal, transverse) in medical images and physical models.
2. Students will compare and contrast computed tomography medical imaging studies of various objects with the actual cross‐sections they create, understanding the function and structure revealed by different imaging techniques.
3. Students will recognize that scientists do critical work in healthcare, such as developing medical imaging technologies to visualize internal structures without invasive procedures.

### 1.2 Lesson structure

The lesson was designed as a part of an interactive, science‐based summer camp experience, “Camps for Curious Minds,” through the Pacific Science Center (Seattle, WA, USA). The general Camps for Curious Minds program is composed of in‐person programing at a variety of locations throughout the greater Seattle area, for students with grade levels ranging from PreK to 8th grade. This lesson was designed to be delivered to two different student groups as a component of two separate week‐long camps: “Young Physicians” for 4th–5th grade students, and “Dissection Lab,” for 6th–8th grade students. In both camps, students learn about the inner workings of the body's systems. In the Young Physicians camp, students learn about careers in medicine and complete guided activities including making a cast. In the Dissection Lab camp, students practice dissection techniques on specimens including an owl pellet, an earthworm, a sheep eye, and a perch. For both camps, the same medical physics outreach activity was used, but with more advanced vocabulary for the 6th–8th grade cohort. The described activity was planned to last 45 min, with students working in pairs. The activity was presented by medical physicists in classroom environments during the summer of 2024.

### 1.3 Materials

All materials for this lesson plan were chosen to be widely available and low cost to the educator. Groceries of various morphological shapes and compositions were scanned prior to the implementation of the lesson: whole chicken, papaya, crawfish, broccoli, whole fish, onion, butternut squash, tangerine, and banana. Groceries were scanned using a GE LightSpeed RT16 scanner (General Electric Healthcare, Milwaukee, WI, USA) with a technique of 120 kV, 97 mA, 0.625 mm slice thickness, 512 × 512 pixel matrix, and a field of view of 25.0 cm. DICOM data was uploaded to DICOM Library (DICOMLibrary.com, Softneta, Lithuania), a free online repository designed to share anonymized medical images. Anonymized patient images were obtained from eContour (eContour.org, University of California‐San Diego, San Diego) a web‐based contouring platform. Bananas and tangerines were chosen because they can be stored without refrigeration, they are able to be cut through the peels reasonably easily by students without sharp implements, they can be peeled by hand if necessary, and they do not represent common food allergens within the United States, where this activity was delivered.^7^ Educators may choose to scan common objects but re‐acquiring data is not required to deliver the lesson: all scan data are available through DICOM Library's public repository, with links included as supplementary materials to this manuscript. Classroom materials required for the lesson are:

1. Computer, internet access, and projector/screen to display imaging studies
2. Digital repository of scanned objects
3. Loaf (or miniature loaf) of bread
4. Multiple butternut squash cut into different planes
5. Activity worksheet (one per student)
6. Bananas (three per student group) and/or tangerines
7. Plastic serrated knives (one per student group)
8. Paper towels

### 1.4 Lesson components

The lesson began by introducing the medical physicist presenters and explaining that scientists play a critical role in healthcare, including in medical imaging. Students learned that medical imaging is a powerful tool to visualize the internal structures of objects and patients without requiring the use of invasive procedures. A small loaf of bread was sliced to demonstrate that stacking “slices” of data can help healthcare professionals visualize a 3D object. Though students have ready familiarity with slices of bread in one plane, additional cuts were used to show, in a simple geometry, the difference between orthogonal planar views. Next, axial images of an anonymized CT scan of a human head were displayed, and students were asked to identify anatomical landmarks including the patient's eyes, nose, ears, and jaw.

**FIGURE 1:**
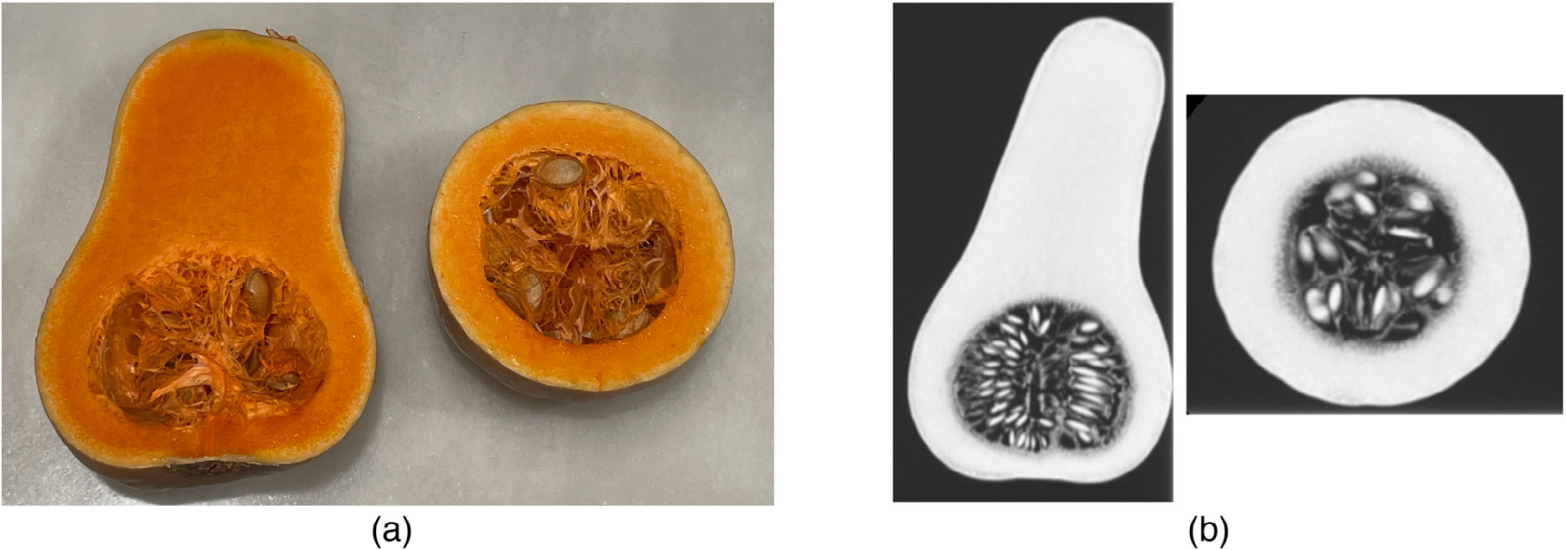
Photographs (a) of butternut squash shown in the lesson plan cut into segments to help students visualize coronal and axial orthogonal planes. Squash was compared to CT images (b) of a similar butternut squash.

Students were then shown CT scan data of a variety of common objects that could be obtained from a grocery store, beginning with a butternut squash. These images, in three orthogonal planes, were compared to multiple squashes that were sectioned to display these planes as visual aids (see Figure 1). The squash had been sectioned in advance for ease of presentation. After comparing the CT data of the squash to the actual sectioned squash, other images were displayed to students, while allowing students to guess the object: whole chicken, papaya, crawfish, broccoli, whole fish, and onion. The CT data was displayed dynamically, with the presenter scrolling through slices and responding to student feedback to go “up” or “down” within any of the orthogonal views.

**FIGURE 2:**
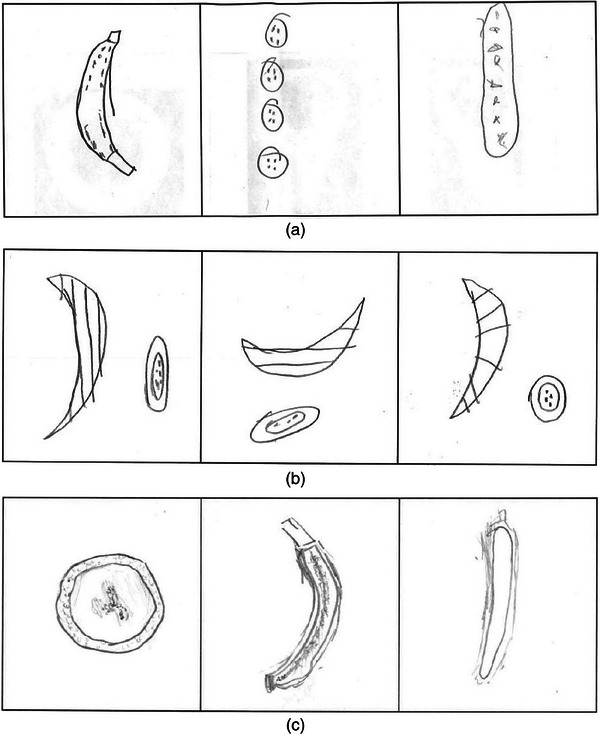
Excerpt of three students’ worksheets, demonstrating students’ response to the hands‐on activity section of the lesson plan. The prompt for this section on the worksheet was, “You are going to cut your bananas carefully into three different planes (use one banana for each plane). Draw a slice, or cross‐section, from each plane below.” Shown in (a) and (b) are example responses from the 4th–5th grade session, and in (c) an example response from the 6th–8th grade session.

Students were grouped into pairs. Each student group was supplied with three bananas and a serrated, disposable plastic knife. A dissection kit could also be used to replace the knife. Each student was supplied with a worksheet to provide guidance and scaffolding through the activity. Students were asked to attempt to predict what multiple orthogonal views of a banana would look like and then to segment the fruit into different planes and record their results on their worksheets. Examples of excerpts of student worksheets are included in Figure 2. Prior to the start of class, camp staff had confirmed that no students had an allergy to bananas (tangerines were also made available in case of a student with an allergy). After students segmented their specimens and completed their worksheets, the CT imaging study of the fruits was displayed.

Following the hands‐on component of the program, summary learning points were reviewed with the students. Each student group was asked to describe any similarities they found with the medical imaging studies activity compared to the dissections they had done earlier in the camp experience. Finally, students were invited to ask any additional questions about the activity, medical imaging, and about medical physics careers in general. A breakdown of the itinerary of the lesson is included in the following section as a suggested timeline.

### 1.5 Lesson outline

1. 10 min: introduction
  - Students are introduced to medical imaging and medical physics.
  - A human patient CT of the head is used to demonstrate the importance of CT.
  - Sliced bread demonstrates how to represent a three‐dimensional object using a series of two‐dimensional images.
2. 10 min: planes and cross sections
  - A CT of a butternut squash is compared to a physical sectioned squash.
  - Students review CT datasets of common groceries.
3. 20 min: hands‐on fruit dissection
  - Students describe what they observe when cutting along the sagittal, coronal, and axial planes.
  - Students compare their recorded cross‐sections with presented imaging studies.
4. 5 min: wrap up and discussion

## 2 DISCUSSION

This lesson was delivered to 12 students for the 4th–5th grade student group, and 15 students for the 6th–8th grade student group in August 2024. Both age ranges initially struggled to identify the head and neck patient CT dataset when scrolling through axial images starting in the neck and scrolling in the superior direction. Both cohorts did identify that they were viewing a head when shown the entire scan. When asked about their reasoning, they pointed out the anatomical landmarks of the patient's eyes. Other basic anatomical features that were discussed by the educators, after students had identified that they were viewing a head CT, included the nose, ears, teeth, and skull. The hands‐on nature of the activity fostered high levels of participation and enthusiasm among students.

One area that some students found challenging, in both cohorts but especially the younger cohort, was the difference between sagittal and coronal planes for the banana, if the axial plane is defined as perpetual to the long axis of the banana. This can be seen in Figure 2b, in which a student from the 4th–5th grade cohort rotated the position of the banana rather than the position of the imaging planes, and used parallel, rather than perpendicular, planes to define segmentation for sagittal and coronal planes. Nevertheless, based on both in‐class, real‐time feedback, and student‐written responses on the scaffolded worksheet, students demonstrated understanding of sectional imaging and its utility in medical imaging, with comparisons between real‐life cross‐sections and the imaging studies reinforcing understanding of how medical imaging techniques provide insight into the human body. For educators repeating this activity, we believe based on our experience that it may be worthwhile, even with a condensed time frame such as the 45‐min time slot, to spend additional time reviewing the difference between sagittal and coronal orientations.

Feedback from both students and the camp content specialist was positive, including comments such as: “I wholeheartedly believe that you are inspiring the next generation of scientists every time you join our campers” (camp content specialist, personal communication, Aug 1, 2024). Example free text student worksheet responses are tabulated in the supplementary materials and included “It was cool to see the x‐rays,” “I enjoyed because I got to see CT scans,” and “I learned what medical images do, they are really cool and useful” (student worksheet responses, Aug 8, 2024). Worksheet responses indicated students generally achieved learning objectives of the lesson plan.

Note that using medical images of fruits, vegetables, and other food products for educational purposes is not new^8^, ^9^, ^10^; however, this lesson plan was designed as a very basic introduction to medical imaging for a young audience as a way to encourage exploration of spatial reasoning and learn about different science career opportunities. The lesson plan makes resources available including the CT datasets, as an accessible means to outreach for interested members of the medical physicist and radiation oncology community. Additionally, while existing resources such as the IOP Medical Physics Group lesson plans^11^ offer in‐depth educational tools for older students, this particular lesson plan is designed to be a brief, introductory activity tailored to younger audiences.

Challenges for this activity included managing time. A 45‐min segment is a short duration for a lesson plan that includes a hands‐on activity, where educators ideally would dedicate a large fraction of the total schedule to the hands‐on components. To manage time effectively, it is recommended to staff the activity with adequate teachers to help encourage students to stay on task during the hands‐on activity and maintain a positive learning environment with minimal disruptive behavior.

Educators who are interested in implementing similar lesson plans in their own communities may wish to incorporate additional imaging modalities. This case study focused solely on CT imaging based on its ease of accessibility to the authors and the brevity of the allotted time for the activity. Educators could also explore the incorporation of oblique slicing as well as modalities such as MRI, PET, and ultrasound. Building on the foundation described by Santoso et al.^8^ live imaging demonstrations could be taken into the classroom, such as the scanning of a gelatin phantom with an embedded grape using a mobile ultrasound system. Note that adding these elements will likely significantly increase the activity time, so educators will need to plan accordingly.

If physicists are integrating a single medical physics lesson plan into existing coursework, collaboration with the regular classroom teachers will help to dovetail activities with existing curricular objectives and ensure that the lesson plan reinforces broader educational goals.

## 3 CONCLUSION

This educational case study highlights the effectiveness of using hands‐on activities and real medical images to teach middle school students about medical imaging. By integrating accessible, interactive elements, students were encouraged to take an active approach to learning, facilitating understanding of different spatial planes, their application in medical imaging, and careers in medical physics. Positive feedback from students and teaching staff indicated that low‐cost, widely accessible materials can be used to enhance comprehension aligning with NGSS. Medical physicists interested in adapting this straightforward lesson plan may decide to incorporate alternative imaging modalities or to reuse the same CT datasets that are available as supplementary materials.

## AUTHOR CONTRIBUTIONS

Roles are defined according to Contribution Roles Taxonomy (CRediT). All authors were responsible for writing (review and editing) and investigation. In addition, the first author was responsible for writing (original draft), conceptualization, resources, methodology, supervision, and project administration. The second author was responsible for data curation.

## CONFLICT OF INTEREST STATEMENT

The authors declare no conflicts of interest.

## Supporting information



SUPPORTING INFORMATION

SUPPORTING INFORMATION

SUPPORTING INFORMATION
