# Supplementary material for: Engaging grade school learners with an interactive medical imaging activity
Source: J Appl Clin Med Phys. 2024 Dec 16;26(1):e14606. doi: 10.1002/acm2.14606 (PMC11713928; doi:10.1002/acm2.14606)
Supplement: Supplementary file 1 — SUPPORTING INFORMATION [file ACM2-26-e14606-s001.pdf]

- Chicken: https://www.dicomlibrary.com?study=1.3.6.1.4.1.44316.6.102.1.2024063081735690.874373137852206207686
- Papaya: https://www.dicomlibrary.com?study=1.3.6.1.4.1.44316.6.102.1.20240630161548156.86763376639850029926
- Orange: https://www.dicomlibrary.com?study=1.3.6.1.4.1.44316.6.102.1.20240630192119332.76450212804300444844
- Banana: https://www.dicomlibrary.com?study=1.3.6.1.4.1.44316.6.102.1.2024063020151176.474156996437023222746
- Crawfish: https://www.dicomlibrary.com?study=1.3.6.1.4.1.44316.6.102.1.2024063016338379.677411058761412999210
- Broccoli: https://www.dicomlibrary.com?study=1.3.6.1.4.1.44316.6.102.1.2024063017144454.909165465732500227816
- Fish: https://www.dicomlibrary.com?study=1.3.6.1.4.1.44316.6.102.1.20240630144848518.95612470894068211184
- Avocado: https://www.dicomlibrary.com?study=1.3.6.1.4.1.44316.6.102.1.2024063019837262.438056436338394375312
- Butternut squash: https://www.dicomlibrary.com?study=1.3.6.1.4.1.44316.6.102.1.20240630184652173.74920939377399754350
- Onion: https://www.dicomlibrary.com?study=1.3.6.1.4.1.44316.6.102.1.20240630172246250.16626219622636428583
