# Supplementary material for: Engaging grade school learners with an interactive medical imaging activity
Source: J Appl Clin Med Phys. 2024 Dec 16;26(1):e14606. doi: 10.1002/acm2.14606 (PMC11713928; doi:10.1002/acm2.14606)
Supplement: Supplementary file 2 — SUPPORTING INFORMATION [file ACM2-26-e14606-s002.pdf]

“Dissection” Medical Imaging Lab

Name: ___________________________________

Below is a picture that is part of a person’s CT scan. The picture was taken with the person lying down with their arms raised. Can you draw arrows from the labels to the picture to show what you think might be the person’s neck, arms, lungs, and arms?

Neck

Arms

Lungs

Ribs

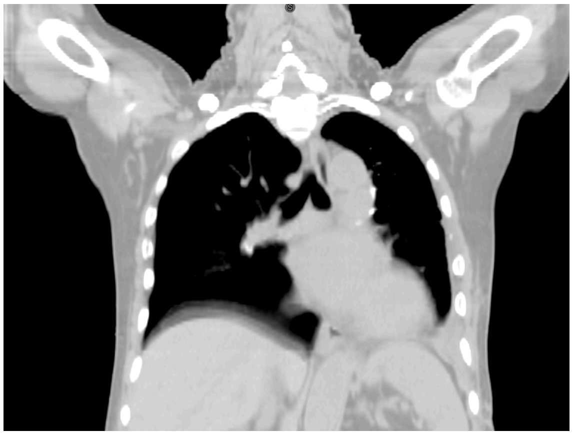

Look at the CT images of the fish and compare them to what you saw when you dissected the fish in class. What parts of the structure do you recognize?

____

____

Sometimes it is helpful in medical imaging to look at different orientations or planes of view of the pictures, the way we cut into loaves of bread differently in class. Below is an example of three different planes of view for a butternut squash. Look at the butternut squash in class. Can you see the similarities between the real squash and the planes shown below?

____

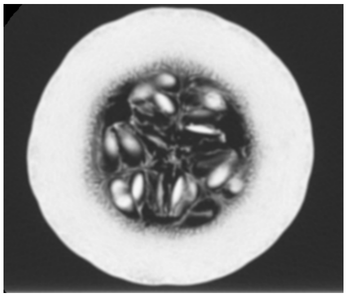	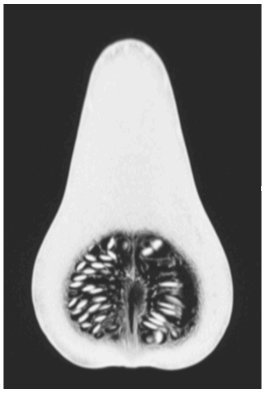	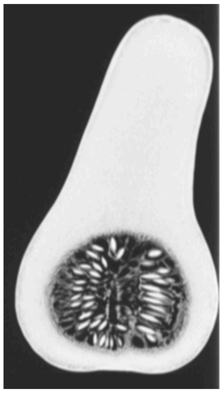

You are going to cut your bananas carefully into three different planes (use one banana for each plane). Draw a slice, or cross section, from **each** plane below.

What are some ways you think medical imaging can be useful?

____

____

Why are scientists important in healthcare?

____

____

Did you enjoy this activity? Why or why not?

____

____
