# Supplementary material for: Engaging grade school learners with an interactive medical imaging activity
Source: J Appl Clin Med Phys. 2024 Dec 16;26(1):e14606. doi: 10.1002/acm2.14606 (PMC11713928; doi:10.1002/acm2.14606)
Supplement: Supplementary file 3 — SUPPORTING INFORMATION [file ACM2-26-e14606-s003.pdf]

Responses from “Dissection Lab” students to the prompts “What are some ways you think medical imaging can be useful?” and “Did you enjoy this activity? Why or why not?”

What are some ways you think medical imaging can be useful?

1. Can observe what problems the human body has, so as to better cure. Also can look at organisms from many angles to know it better.
2. Its useful because you dont have to cut into the person
3. to see internal things without cutting them
4. to find cancer
5. [no answer]
6. Used to treat diseases and find problems in body
7. The ways I think medical imaging can be useful is the front, middle, bottom, and top.
8. Different angels of images makes it easier to find out the problem in the patient’s body and how to make them feel better.
9. So doctors dont have to constantly people open
10. to see stuff without cutting into it
11. So you can help humens and so you can mack sher that you don’t miss enething.
12. seeing what’s going on inside someone’s body without cutting it up.
13. You can see the inside of items without cutting it open
14. [illegible]
15. [no answer]

Did you enjoy this activity? Why or why not?

1. Yes. It is interesting and I can learn a lot of knowledge.
2. I enjoyed this because I could cut in a banana
3. Kind of. It was fun but all lot of writing
4. yeah, Because it was easy and not boring, and short.
5. [no answer]
6. Yes. It was cool to see the x-rays
7. Yes. Because I like when we were doing the banana, like seeing all stuff we can find all information, and scientific things we see.
8. Yes, I learned what medical images do, they are really cool and useful
9. Yes
10. yes it was hands on
11. It was fun!
12. It was nice enough, I liked cutting up the banana.
13. I enjoyed because I got to see ct scans
14. Yes [illegible]
15. yes
